# Application of Chitosan@Fe_3_O_4_ Nanoparticle-Modified Screen-Printed Graphene-Based Electrode for Simultaneous Analysis of Nitrite and Ascorbic Acid in Hydroponics and Fruit Juice

**DOI:** 10.3390/s25051431

**Published:** 2025-02-26

**Authors:** Sudarut Pitakrut, Phetlada Sanchayanukun, Chanpen Karuwan, Sasithorn Muncharoen

**Affiliations:** 1 Department of Chemistry, Faculty of Science, Burapha University, Chonburi 20130, Thailand; sudarutsg@gmail.com (S.P.); phetrlada.sa@go.buu.ac.th (P.S.); 2 National Nanotechnology Center (NANOTEC), National Science and Technology Development Agency (NSTDA), Pathum Thani 12120, Thailand; chanpen.kar@nanotec.or.th

**Keywords:** magnetic nanoparticles, chitosan, SWV, hydroponics, food

## Abstract

In this work, the development of screen-printed electrodes modified with chitosan-coated magnetite nanoparticles (CTS@Fe_3_O_4_/SPGNE) for the simultaneous determination of nitrite (NO_2_^−^) and ascorbic acid (AA^−^) is presented. The study investigated various ratios of graphene to chitosan-coated magnetite nanoparticles (CTS@Fe_3_O_4_), as well as the optimal pH. These factors were examined due to their impact on the selectivity and sensitivity of the analysis. The results indicated that a graphene paste to CTS@Fe_3_O_4_ ratio of 16:1.0 g and a pH of 4 were optimal for the analysis of both NO_2_^−^ and AA^−^. Additionally, the behavior of the proposed electrode, its analytical performance, and interference studies were thoroughly examined. Furthermore, the CTS@Fe_3_O_4_/SPGNE electrode shows potential for the simultaneous determination of NO_2_^−^ and AA^−^ in hydroponics and fruit juice samples.

## 1. Introduction

In the present, maintaining a healthy body is crucial, particularly in the context of the increasing prevalence of various diseases. Vitamins are widely recognized as essential nutrients that play an important role in disease prevention. Among these, ascorbic acid (AA^−^), commonly referred to as vitamin C, is vital for supporting the immune system’s normal functioning, thereby enhancing the body’s ability to combat illnesses. Notably, the human body cannot synthesize AA^−^ endogenously and must rely on dietary sources to meet its requirements. However, adequate AA^−^ is obtained from the diet; therefore, additional supplementation is unnecessary and may even pose potential side effects. As already known, vegetables and fruits are primary sources of AA^−^, with fruits offering a particularly high content of this essential nutrient. However, fruit consumption also introduces a considerable amount of sugar, which may pose challenges for individuals managing their sugar intake. Consequently, vegetables are a preferable alternative, as they provide adequate AA^−^ with lower sugar content while also contributing dietary fiber that supports healthy digestion. It is important to note that AA^−^ is an unstable compound, and its content can be significantly diminished during the cooking process. Currently, the Institute of Medicine in USA recommends a daily intake of AA^−^ of 75 mg per day for women and 90 mg per day for men [1], while the Institute of Medicine in Thailand recommends 85 mg per day for women and 100 mg per day for men [2]. A deficiency in AA^−^ can lead to a variety of health issues, including scurvy, increased susceptibility to colds, and recurrent infections. Furthermore, prolonged AA deficiency may result in fragile capillaries, easy bruising, muscle weakness, and bone pain [3,4,5]. Consequently, the consumption of fresh vegetables is an effective means of ensuring adequate vitamin C intake, especially for diabetic people.

The consumption of fresh vegetables has gained increasing popularity, including those grown using soil-based and soilless systems, such as hydroponics. Recently, hydroponic vegetable cultivation has attracted significant attention due to its efficient use of limited cultivation space, simplified management of weeds and pests, and high market value. In hydroponic farming, plants receive nutrients in the form of nutrient solutions, which must be sufficiently concentrated to support plant growth. These nutrients are directly absorbed by the plants. For fresh vegetable consumption, nitrogen (N) is typically supplied to promote growth, enhance leaf size, and produce a dark green, appealing appearance. This nitrogen is often provided in the form of urea fertilizer solution. The application of urea solutions involves key chemical processes, including ammonification, which converts urea into ammonia, and nitrification, which further converts ammonia into nitrite and nitrate. Thus, the direct absorption of nutrient solutions in hydroponics can increase the accumulation of chemical residues, particularly nitrite (NO_2_^−^), in various plant parts. In nature, the reversibility of NO_2_^−^ and NO_3_^−^ can occur in living organisms. For plant NO_2_^−^ transport, it has been reported that the cytosol is the first cellular compartment to encounter NO_3_^−^ in the root, where it is converted to NO_2_^−^ as cytosolic NO_2_^−^. This product can then be transported across the membrane, allowing NO_2_^−^ to be detected in plants [6]. Additionally, NO_2_^−^ can be used as an additive in fruit juice. Therefore, NO_2_^−^ was selected as the analyte for this study. As known, NO_2_^−^ is a potent oxidizing agent that inhibits hemoglobin from transporting oxygen to body tissues, leading to conditions such as fatigue, cyanosis—commonly known as blue baby syndrome—infections, diabetes, cancer, and cerebrovascular diseases [7,8,9,10,11]. The Ministry of Public Health in Thailand has set the standard for NO_2_^−^ levels in food to not exceed 80 mg·kg^−^^1^, and the amount consumed by the body should not exceed 0.07 mg·kg^−^^1^ of body weight per day [12]. Therefore, the simultaneous analysis of NO_2_^−^ and AA^−^ content in hydroponic vegetables and fruit juice has garnered considerable interest, with both used as samples in this work. Various techniques, such as UV–Visible spectrophotometry, High-Performance Liquid Chromatography (HPLC), and Gas Chromatography (GC), have been used to analyze NO_2_^−^ and AA^−^. However, research on their simultaneous analysis using these methods is limited. Only a few studies have focused on electrochemical methods for the simultaneous detection of these compounds [13,14,15,16,17,18,19,20].

To exploit the advantages of screen-printed electrodes (SPEs) and build on our research group’s previous work on CTS@Fe_3_O_4_ particles [21,22,23] and graphene-based screen-printing ink [24], this study aims to fabricate a novel CTS@Fe_3_O_4_/SPGNE. The aim is to enhance specificity and sensitivity for the simultaneous determination of NO_2_^−^ and AA^−^ in hydroponic vegetables and fruit juice samples, ensuring the food quality meets the established safety standards.

## 2. Materials and Methods

### 2.1. Apparatus

All electrochemical experiments in this work were performed using the potentiostat (Autolab PGSTAT204, Metrohm, The Netherlands) and operated via Nova version 1.11 software). A ball-mill machine (Ball milling Emax, Rutsch, UK) was used to screen the three-electrode system. A screen printer (Dek, version 03ix, UK) was employed to screen-print the conductive inks on individual PET substrates. The three-system screen-printed electrode was produced by mixed CTS@Fe_3_O_4_ and graphene paste used as the working and auxiliary electrodes, while the silver/silver chloride paste (Ag/AgCl) was screened and used as a reference electrode. A specord 210 plus UV–Visible spectrophotometer (Analytica Jana/Germany) was used for the determination of the analytes used as the standard method. A scanning electron microscope (SEM) and energy-dispersive X-ray spectrophotometer (EDX) (LEO 1450 VP; Carl Zeiss) were employed to observe the surface morphologies and elemental analysis of graphene and CTS@Fe_3_O_4_/graphene. Additionally, the FT-IR spectra of graphene and CTS@Fe_3_O_4_/graphene were obtained by using an FT-IR spectrophotometer (Perkin Elemer, Spectrum System 2000).

### 2.2. Synthesis of Magnetite Nanoparticles and Coated Chitosan on Magnetite Nanoparticles

Fe_3_O_4_ nanoparticles were prepared by the co-precipitation method of ferric and ferrous ions using ammonium hydroxide, as described by Sanchayanukun and Muncharoen (2020) [22]. Briefly, it involves mixing 2.0 M ferrous chloride tetrahydrate (Panreac QuÍmica S.A., Spain) and 1.0 M ferric chloride anhydrous (Loba Chemie, India) with a 1:2 ratio in a round-bottomed bottle. While the mixed solution was stirred, 0.7 M ammonium hydroxide (Loba Chemie, India) was dropped for 2 h, after which dark-brown magnetite nanoparticles appeared. After that, the particles were repeatedly rinsed and dried at 120 °C. Then, the Fe_3_O_4_ nanoparticles were cooled at room temperature and stored in desiccator. For CTS@Fe_3_O_4_ nanoparticles’ synthesis, the reverse-phase suspension crosslink method was used. Weigh the Fe_3_O_4_ nanoparticles 0.100× g in a small vial and wash with ethanol. After that, paraffin (Fisher Scientific, USA), Span-80 (Sigma-Aldrich, Germany), and 4% *w*/*v* chitosan (Sigma-Aldrich, Iceland) were added, respectively. The mixture was sonicated for 30 min. Next, 25% glutaraldehyde (Loba Chemie, India) was added and shaken using a shaker (DLAB, China) for 4 h. At the end of time, the CTS@Fe_3_O_4_ nanoparticles were obtained. The synthesized CTS@Fe_3_O_4_ nanoparticles were separated from the unreacted chitosan solution under magnetic field. Subsequently, the CTS@Fe_3_O_4_ nanoparticles were repeatedly washed with distilled water and ethanol before being filtered. The synthesized particles were dried in the oven at 50 °C for 12 h. Later, the CTS@Fe_3_O_4_ nanoparticles were stored in a desiccator before use.

### 2.3. Preparation of CTS@Fe_3_O_4_ Nanoparticles Modified Screen-Printed Graphene-Based Electrode (CTS@Fe_3_O_4_/SPGNE)

Firstly, graphite rods (1/400 dia, Electron Microscopy Science) were used as the starting material for graphene synthesis. Graphene powder was synthesized by one-step electrolytic exfoliation in a PSS solution, based on similar procedures reported previously [24] using the electrolytic exfoliation method. A commercial polystyrene sulfonic acid (PSS) solution (Clevios P Jet N from HC Starck, USA) was employed as an electrolyte for electrolytic exfoliation. A constant voltage of 8 V was applied between two graphite rods placed in an electrolysis cell with the PSS electrolyte for 24 h. The graphene powder was then extracted from the solution by washing ethanol and deionized water several times and drying at 80 °C for 1 h. The graphene powder was obtained.

Secondly, to prepare the graphene paste, 10% graphene powder was carefully blended with a commercial carbon paste using a ball-mill machine for 1 h to ensure a homogeneous mixture.

Lastly, the CTS@Fe_3_O_4_/SPGNE conductive ink was prepared by mixing the prepared graphene paste and the synthesized CTS@Fe_3_O_4_ nanoparticles in a weight ratio of 16:1 by using a ball mill at 800 rpm for 1 h. The CTS@Fe_3_O_4_/SPGNE conductive ink was then screened on a polyvinyl chloride (PVC) substrate to produce working and counter electrodes. The printed substrate was then dried at 60 °C for 5 min. This was followed by the screen printing of Ag/AgCl ink as the reference electrode. Finally, polyurethane ink was screened over the three electrodes to define the sensing area.

### 2.4. Square-Wave Voltametric Method

The optimum conditions for square-wave voltammetry (SWV) were carried out in a 0.1 M phosphate-buffer solution containing pH 4. A pre-concentration step at potential −0.5 V for 15 s, scan potential −0.5 to 1.2 V, frequency 25 Hz, amplitude 25 mV s^−1,^ and step potential 10 mV s^−1^ were used as the optimum voltametric parameters. The results of the study of the optimum conditions for SWV parameters were shown in Supplementary Figure S1.

### 2.5. Sample Preparation

At a local market in Bang Saen, Chonburi, Thailand, fruit juice and hydroponic vegetable samples were chosen and acquired for use in this study. The preparation method for the fruit juice samples was adopted from Pisoschi et al. (2011) [25]. The fruit juice samples were filtered using no. 1 filter paper. Following this, the resulting solution underwent centrifugation at 10,000 rpm for 5 min. Afterward, the sample solutions were stored in a refrigerator at 4 °C. The hydroponic vegetable samples were prepared following the guidelines of the international standard methods (International Standard ISO 6335, 1984) [26] and Stachniuk et al. (2018) [27]. Following the weighing of the 10 g sample, successive rinses with tap water and deionized water were conducted, after which the sample underwent coarse and fine grinding using a blender. After the addition of sodium tetraborate and warm water, the finely ground sample was boiled for 15 min. Following this step, potassium hexacyanoferrate and zinc acetate were introduced. The solution was then filtered using no.1 filter paper and centrifuged at 10,000 rpm for 5 min. These samples were ultimately examined using the optimal conditions for both methods: UV–Visible spectrophotometry as the standard method, and the developed method utilizing electrochemistry.

## 3. Results and Discussion

### 3.1. Characterization

To characterize the materials, CTS@Fe_3_O_4_ mixed with graphene was used as the material for the proposed electrode. Therefore, SEM/EDX was employed for morphology and elemental analysis, while the FT-IR technique was used to identify the materials. Additionally, electrochemical impedance spectroscopy (EIS) was used to evaluate the material’s conductivity performance.

#### 3.1.1. SEM/EDX

**Figure 1:**
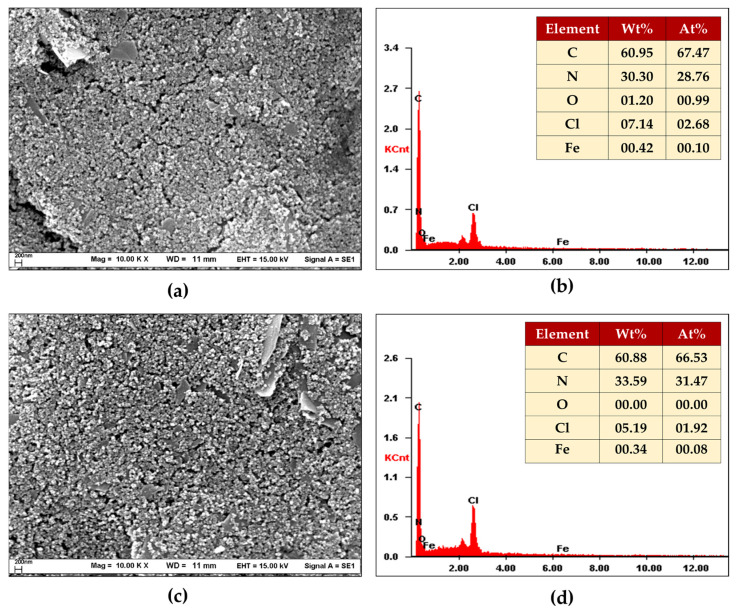
Scanning electron microscopy (SEM) images study of (**a**) graphene and (**c**) CTS@Fe_3_O_4_/graphene at 10,000× magnification; and EDX composition study of (**b**) graphene and (**d**) CTS@Fe_3_O_4_/graphene.

The results, depicted in Figure 1, show that the %wt of the elements in graphene and CTS@Fe_3_O_4_/graphene were slightly different. However, when considering the %wt of nitrogen (N), the %wt of N in CTS@Fe_3_O_4_/graphene (33.59 %wt) was higher than that in graphene (30.30 %wt). To further validate the hypothesis, the FT-IR technique was employed for further analysis.

#### 3.1.2. FT-IR

**Figure 2:**
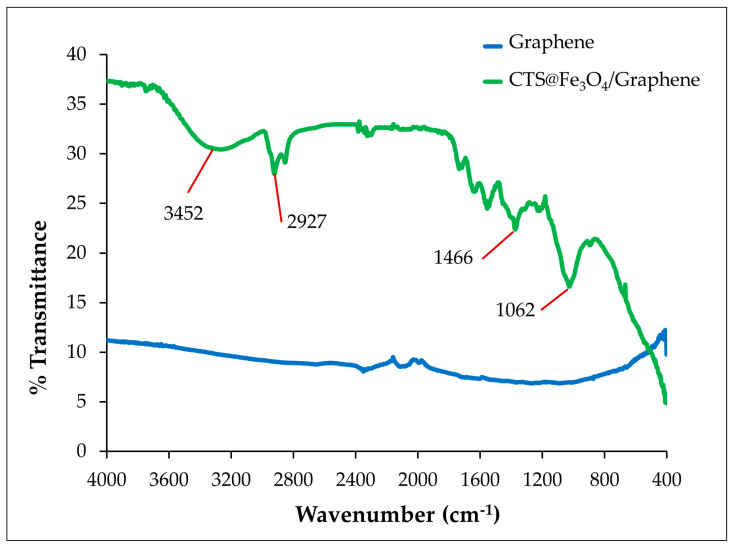
FT-IR spectra of graphene (blue line) and CTS@Fe_3_O_4_/graphene (green line).

The composition of graphene and CTS@Fe_3_O_4_/graphene was analyzed using FT-IR spectroscopy to confirm the successful incorporation of CTS@Fe_3_O_4_ into graphene as the proposed electrode material. Figure 2 represents the FT-IR spectrum of these materials. In the case of CTS@Fe_3_O_4_/graphene, the peak at 3452 cm^−1^ is attributed to the O-H and N-H stretching vibrations, while the peak at 2927 cm^−1^ corresponds to C-H stretching vibrations. Additionally, the peak at 1062 cm^−1^ is associated with C-O stretching vibrations in chitosan [28]. The peak at 1466 cm^−1^ also corresponds to the -NH_2_ group resulting from the crosslinking reaction between chitosan and glutaraldehyde, as reported by Bin Li et al. [29]. For bare graphene, no FT-IR peaks were observed. Thus, the proposed electrode confirmed successful fabrication using a mixture of CTS@Fe_3_O_4_ and graphene paste.

#### 3.1.3. Electrochemical Impedance Spectroscopy (EIS)

**Figure 3:**
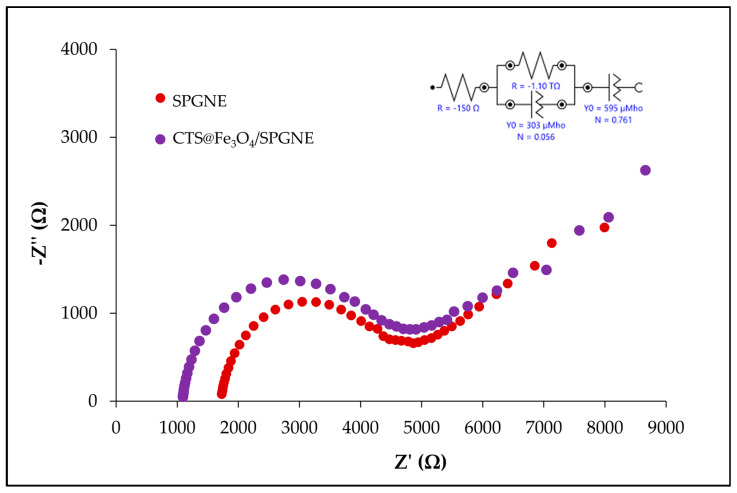
Nyquist diagrams of SPGNE (red line) and CTS@Fe_3_O_4_/SPGNE (purple line) for K_4_Fe(CN)_6_ analysis.

The electrical conductivity of the SPGNE and CTS@Fe_3_O_4_/SPGNE were studied using EIS technique. The Nyquist diagram usually consists of two parts: a semicircle illustrating an electrode’s electrical conductivity. The narrower hemisphere denotes strong conductivity or low resistance. Additionally, there is a straight line depicting the electrode’s diffusion process. In Figure 3, the Nyquist diagrams illustrate the analysis of K_4_Fe(CN)_6_ using these electrodes. The outcomes revealed that the SPGNE (red line) exhibited superior electrical conductivity, attributed to the inherent conductivity of graphene. Subsequently, CTS@Fe_3_O_4_/SPGNE (purple line) demonstrated lower conductivity, attributed to the non-conductive polymer chitosan coating the surface of the magnetite nanoparticles. This coating, as observed in studies by Marroquin et al. and Abedi et al. [30,31], obscures the conductivity of graphene, resulting in lower conductive nature of this electrode type. Nevertheless, the sensitivity observed in the analysis of NO_2_^−^ and AA^−^ using CTS@Fe_3_O_4_/SPGNE surpassed that of SPGNE. This could potentially be attributed to the magnetite nanoparticles coated with chitosan, enhancing adsorption at the newly developed electrode surface, as indicated in the findings reported by Sanchayanukun and Muncharoen (2020) [22]. Thus, CTS@Fe_3_O_4_/SPGNE was chosen and subsequently utilized for further analysis and description.

### 3.2. Preliminary Investigations for Nitrite (NO_2_^−^) and Ascorbic Acid (AA^−^) Analysis

**Figure 4:**
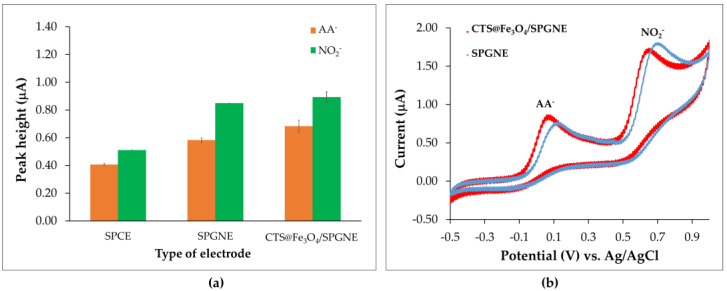
(**a**) The relation plot between peak height (current) and various material electrode types and (**b**) cyclic voltammograms for the analysis of nitrite (NO_2_^−^) 0.05 mM and ascorbic acid (AA^−^) 0.1 mM in phosphate-buffer solution (pH 4) detected by CTS@Fe_3_O_4_/SPGNE (red line) and SPGNE (blue line).

Various electrode types—screen-printed graphite electrodes (SPCE), screen—printed graphene electrodes (SPGNE), and CTS@Fe_3_O_4_/SPGNE—were used for the simultaneous analysis of NO_2_^−^ and AA^−^ in this study. The relation plot between current (peak height) and electrode types is shown in Figure 4a. It was observed that the oxidation currents of NO_2_^−^ and AA^−^ detected by CTS@Fe_3_O_4_/SPGNE gave the highest compared to those detected by SPCE and SPGNE. The oxidation reactions of NO_2_^−^ and AA^−^ were shown in Supplementary Figure S2. Additionally, it was also found that the potential of NO_2_^−^ (at 0.65 V) and AA^−^ (at 0.08 V) detected by CTS@Fe_3_O_4_/SPGNE was slightly lower than the potential of NO_2_^−^ (at 0.70 V) and AA^−^ (at 0.14 V) detected by SPGNE (Figure 4b). This could be attributed to the robust electrical conductivity and catalytic properties exhibited by Fe_3_O_4_ nanoparticles, along with the attraction between the positive charge of amine in chitosan and the analytes (NO_2_^−^ and AA^−^). This aligns with findings from prior research by Mo et al. [32] and Sanchayanukun and Muncharoen (2020) [22]. Consequently, CTS@Fe_3_O_4_/SPGNE was selected as the suitable electrode for further experiments. The cyclic voltammograms of NO_2_^−^ and AA^−^ compared to a blank signal were shown in Figure 4b. Additionally, the cyclic voltammograms of phosphate buffer (pH 4) recorded using SPGNE exhibited a higher background current, as shown in Figure S2 (Supplementary Figure S2). Furthermore, the simultaneous analysis of NO_2_^−^ and AA^−^ using the SWV technique with the SPGNE and CTS@Fe_3_O_4_/SPGNE was investigated. The SWV voltammograms obtained using CTS@Fe_3_O_4_/SPGNE exhibited suitable response signals, as illustrated in Figure S2 (Supplementary Figure S2). The surface areas of the modified (CTS@Fe_3_O_4_/SPGNE) and unmodified (bare SPGNE) electrodes were calculated using the Randles–Ševčík equation [33]. The electroactive surface areas were determined to be 0.2921 mm^2^ for bare SPGNE and 0.3424 mm^2^ for CTS@Fe_3_O_4_/SPGNE, respectively. Thus, the CTS@Fe_3_O_4_/SPGNE was chosen as the three-electrode system for this work.

### 3.3. Ratio of Graphene and Chitosan-Coated Magnetite Nanoparticles (CTS@Fe_3_O_4_)

**Figure 5:**
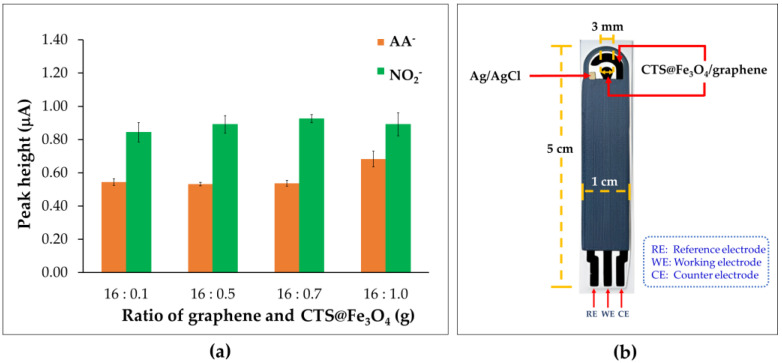
(**a**) The relation plot between peak height (current) and the various CTS@Fe_3_O_4_/SPGNE using different weight ratios of graphene paste and CTS@Fe_3_O_4_ for NO_2_^−^ and AA^−^ analysis and (**b**) the scheme of CTS@Fe_3_O_4_/SPGNE.

The various weight ratios of graphene paste and CTS@Fe_3_O_4_, such as 16:0.1, 16:0.5, 16:0.7, 16:1.0, and 16:1.5 for NO_2_^−^ and AA^−^ analysis were investigated in this study. The results showed that CTS@Fe_3_O_4_/SPGNE could not be prepared at a weight ratio of 16:1.5, as it did not form a paste. Therefore, this ratio was unsuitable for electrode fabrication. For others, the observed oxidation currents of NO_2_^−^ were a bit different. However, it was discovered through the analysis of AA^−^ that the optimum weight ratio between graphene paste and CTS@Fe_3_O_4_ was 16:1.0 (Figure 5). Therefore, the proposed electrode fabricated using the ratio of graphene paste and CTS@Fe_3_O_4_ at 16:1.0 g was selected for the determination of NO_2_^−^ and AA^−^. The scheme of CTS@Fe_3_O_4_/SPGNE was shown in Figure 5b.

### 3.4. Behavior Study of the CTS@Fe_3_O_4_/SPGNE

**Figure 6:**
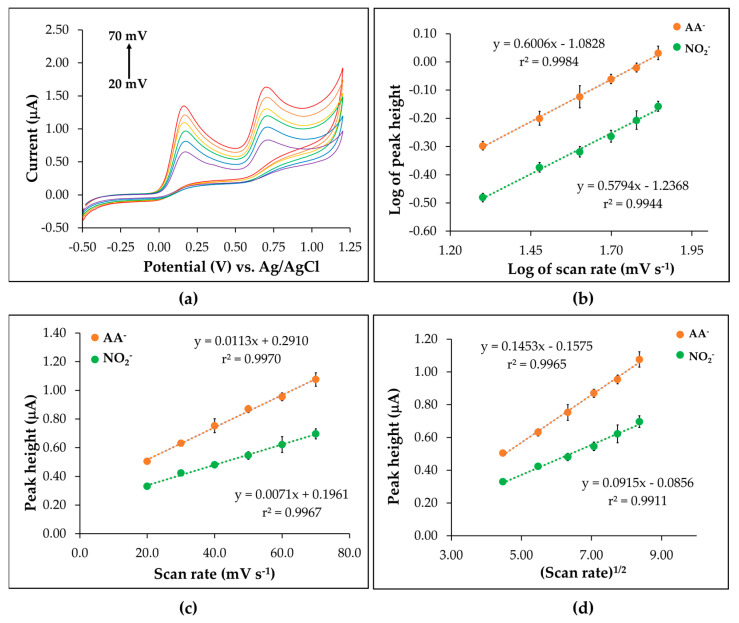
(**a**) Cyclic voltammograms of AA^−^ and NO_2_^−^, (**b**) plots of log peak height and log scan rate, (**c**) plots of peak height and scan rate, and (**d**) plots of peak height and (scan rate)^1/2^. Orange lines represent AA^−^, and green lines represent NO_2_^−^ (**b**–**d**).

The behavior of the developed screen-printed electrode (CTS@Fe_3_O_4_/SPGNE) was studied using the CV technique at a scan rate ranging from 20 to 70 mV. Figure 6a illustrates that the acquired signal rises proportionally with the scan-rate increment. Here, an examination was carried out to explore the adsorption and diffusion behavior through an investigation into the electrode’s oxidation current. Referring to Figure 6b, the linearity range slopes for NO_2_^−^ and AA^−^ determine the electrode behavior. When the slope measures below 0.5, the electrode behavior signifies a diffusion process; if it falls between 0.5 and 1.0, it indicates a mixed diffusion and adsorption process. Slopes exceeding 1.0 imply an adsorption process [34]. The obtained results revealed slopes of 0.6006 for NO_2_^−^ and 0.5794 for AA^−^, indicating that the electrode behavior provides both diffusion and adsorption for both analytes. Additionally, the peak current changed linearly with the scan rate (Figure 6c), indicating an adsorption effect on the mechanism, consistent with the report by Asangil, D. et al. [34]. Furthermore, the peak current also showed a linear relationship with the square root of the scan rate (Figure 6d), which may suggest a diffusion effect. These results indicate that the proposed electrodes exhibit a mixed diffusion and adsorption behavior.

### 3.5. pH Effect

**Figure 7:**
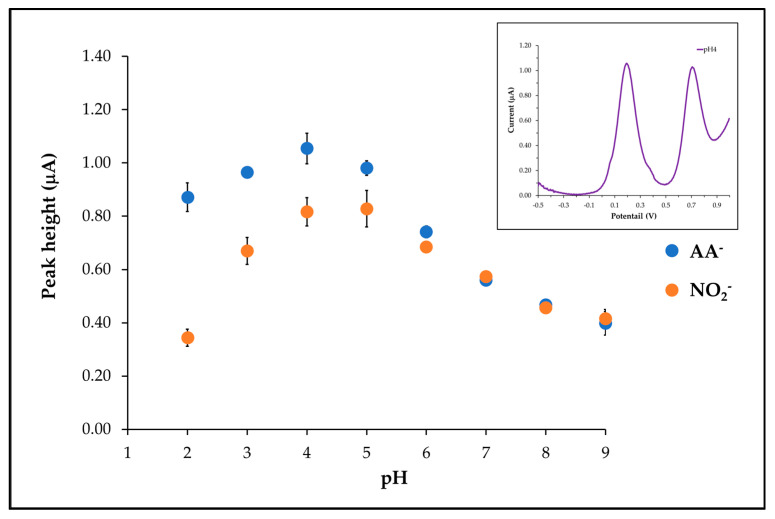
The relation plot between peak height currents of AA^−^ and NO_2_^−^ and various pH of the solutions and (inset) SWV voltammogram of AA^−^ and NO_2_^−^ in phosphate buffer (pH 4).

The electrolyte chosen for the determination of NO_2_^−^ and AA^−^ using the proposed electrode was a phosphate buffer. The results revealed the highest nitrite oxidation current at pH 4 and pH 5. Notably, the standard deviation (s.d.) at pH 4 was lower than that at pH 5. Additionally, pH 4 for AA^−^ provided the highest current response, as shown in Figure 7. The observed effect, corresponding to Sanchayanukun and Muncharoen (2020) and Pitakrut et al. (2023) [22,23], could potentially be explained by the attraction between the positively charged protons originating from the amine functional group (-NH_2_) of chitosan on the nano-magnetite surface and the negatively charged NO_2_^−^ and AA^−^ ions in acidic solutions, leading to the high current. Subsequently, pH 4 was chosen as the suitable electrolyte solution for further NO_2_^−^ and AA^−^ measurement.

### 3.6. Analytical Performance

#### 3.6.1. Linear Range

**Figure 8:**
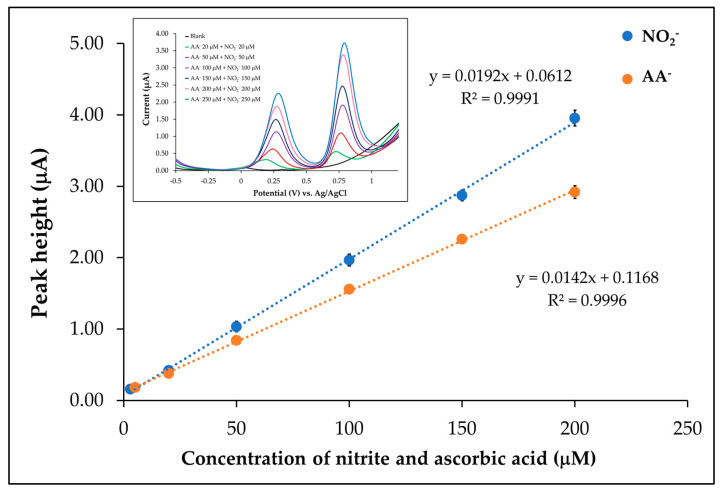
Calibration plots under the optimum conditions for simultaneous determination of AA^−^ (orange line) and NO_2_^−^ (blue line), with inserts displaying square-wave voltammograms.

NO_2_^−^ and AA^−^ at various concentrations were analyzed by the proposed electrode. Under optimum conditions, the linearity of the NO_2_^−^ was 3.00–200.00 µM (y = 0.0192x + 0.0612) with the correlation coefficient (r^2^) of 0.9991 and the linearity of the AA^−^ was 5.00–200.00 µM (y = 0.0142x + 0.1168) with a correlation coefficient (r^2^) of 0.9996. The calibration curves are shown in Figure 8. Additionally, the calibration of NO_2_^−^ was investigated at a fixed concentration of AA^−^. The results showed that the AA^−^ signals were not affected by the increasing concentration of NO_2_^−^. Conversely, the calibration for AA^−^ at a fixed concentration of NO_2_^−^ was also studied. The responses observed that the NO_2_^−^ signals were not influenced by an increasing concentration of AA^−^, as shown in Figure S3 (Supplementary Figure S3).

#### 3.6.2. Limit of Detection (LOD) and Limit of Quantitation (LOQ)

The LOD and LOQ were calculated using 3 s.d./slope and 10 s.d./slope, respectively. For NO_2_^−^, the LOD and LOQ were 2.84 and 9.47 μM, while for AA^−^, the values were determined as 3.39 and 11.30 μM, respectively. These results demonstrate that the proposed method offers high sensitivity and can be applied for the determination of NO_2_^−^ and AA^−^ in real samples, as shown in Table 1.

#### 3.6.3. Repeatability and Reproducibility

In studying the stability of the produced electrodes, two categories were explored. Initially, repeated measurements within the same electrode were conducted, performing five repetitions to acquire the currents for NO_2_^−^ and AA^−^ using CTS@Fe_3_O_4_/SPGNE. The %RSD values were 5.45 for NO_2_^−^ and 4.80 for AA^−^ (*n* = 5). The second aspect concentrated on reproducibility across 10 different electrodes, yielding %RSD values of 4.73 for AA^−^ and 4.00 for NO_2_^−^, detailed in Table 1.

#### 3.6.4. %Recovery

The accuracy of this method was confirmed by assessing %recovery through the analysis of NO_2_^−^ and AA^−^ in food samples. The %recovery values obtained were 80–114% for NO_2_^−^ and 86–115% for AA^−^, as presented in Table 2. These results gave the acceptable criteria, outlined in the AOAC standard [35].

#### 3.6.5. Selectivity

The developed method aimed to analyze NO_2_^−^ and AA^−^ in food products susceptible to contamination by substances like glucose, zinc, urea, nitrate, citric acid, or carbonate. Thus, the interference (IF) was investigated herein. Considered within the limit of tolerance, the signals obtained after adding analyses of these compounds to a solution of NO_2_^−^ and AA^−^ at a concentration of 50.00 M did not exceed 5%, as per Pardakhty et al. (2016) and Ma et al. (2019) [36,37]. As for the results, glucose as the IF exhibited a maximum resistance of 5% at NO_2_^−^:AA^−^:IF ratio of 1:1:500, while for other interfering factors like zinc, urea, and nitrate, the NO_2_^−^:AA^−^:IF ratio was 1:1:50. Furthermore, citric acid and carbonate interferences showed ratios of 1:1:20 and 1:1:5, respectively, as detailed in Table 3. These findings suggest that these interferences do not significantly impact the analysis of NO_2_^−^ and AA^−^.

### 3.7. Application for Food Products

The developed electrodes were utilized for the simultaneous analysis of NO_2_^−^ and AA^−^ in various food samples, including strawberry juice, guava juice, green oak, basil, and laboratory-synthesized samples. The standard addition method was used for all samples. The response signals for NO_2_^−^ and AA^−^ obtained through the proposed method were compared to those from the standard method (UV–Visible spectrophotometric method) as shown in Table 4. It was found that the two methods were not significantly different at the 95% confidence level (t_stat_ = 0.59, t_crit_ = 2.20 NO_2_^−^; t_stat_ = 1.01, t_crit_ = 2.20 AA^−^).

### 3.8. Comparison with Previous Research Work

Table 5 presents a comparison of the proposed method using CTS@Fe_3_O_4_/SPGNE with several studies from the literature. These studies employed various voltametric techniques and different types of working electrodes. It was found that most nanoparticle-modified electrodes exhibited high sensitivity, including the method presented in this work [32,38,39,40,41,42,43,44,45]. However, few studies have investigated the simultaneous analysis of AA^−^ and NO_2_^−^. For fruit juice, the results of this study demonstrated a broader linearity range for the simultaneous determination of the analytes compared to the report by Bartolome, J.P. et al. [45]. Furthermore, this study reveals an appropriate linearity range and sensitivity for the concurrent determination of AA^−^ and NO_2_^−^ in both fruit juice and hydroponic vegetable samples.

## 4. Conclusions

The proposed electrode (CTS@Fe_3_O_4_/SPGNE) demonstrated high performance for the simultaneous determination of NO_2_^−^ and AA^−^. Additionally, mixed behavior (diffusion and adsorption) was observed. Under optimal conditions, the linearity ranges for NO_2_^−^ and AA^−^ were 3.00–200.00 µM and 5.00–200.00 µM, respectively, with limits of detection (LOD) of 2.84 µM and 3.39 µM. The reproducibility of the proposed electrode was confirmed by its precision, showing a relative standard deviation (RSD) of 4.00% for NO_2_^−^ and 4.73% for AA^−^ (*n* = 10). Furthermore, the developed method was successfully applied to the analysis of NO_2_^−^ and AA^−^ in food samples, particularly fruit juice and hydroponic vegetables, demonstrating consistency with the standard method.

## 5. Patents

Patent Application Pending 2303003313 (2023) [46].

## Figures and Tables

**Table 1:** Analytical performance for determination of NO_2_^−^ and AA^−^ under optimal conditions.

Parameter	Results	
	AA^−^	NO_2_^−^
Linear range (µM)	5.00–200.00	3.00–200.00
Linear equation (µM)	y = 0.0142x + 0.1168	y = 0.0192x + 0.0612
Limit of detection: LOD (µM)	3.39	2.84
Limit of quantitation: LOQ (µM)	11.30	9.47
Repeatability (*n* = 5)	4.80	5.45
Reproducibility (*n* = 10)	4.73	4.00

**Table 2:** %Recovery for the determination of NO_2_^−^ and AA^−^ in real samples using the developed method.

Sample	% Recovery (*n* = 3)	
	AA^−^	NO_2_^−^
Strawberry juice	104.41–112.98	94.14–98.71
Guava juice	93.92–114.92	80.19–93.39
Green oak	103.83–107.37	90.89–101.33
Basil	86.22–104.85	82.16–89.43
Synthetic samples	107.37–114.77	96.97–114.05
Firilice	97.38–106.23	87.51–98.22
Bok choy	95.55–110.29	90.75–100.03
Cos	92.25–101.90	83.53–87.60
Red oak	83.28–86.13	88.12–99.64
Butterhead lettuce	92.83–106.17	95.59–106.36
Red coral	93.62–105.24	98.44–102.25
Hydroponic nutrient	88.57–96.82	89.74–96.18

**Table 3:** %Tolerance for signals of NO_2_^−^ and AA^−^ under various disturbances.

Interference	Concentration Ratio			%Tolerance (*n* = 3)	
	AA^−^	NO_2_^−^	IF ^a^	AA^−^	NO_2_^−^
Glucose	1	1	500	2.73	4.91
Zinc	1	1	50	4.73	2.59
Urea	1	1	50	1.67	1.31
Nitrate	1	1	50	1.86	0.15
Citric acid	1	1	20	4.78	1.86
Carbonate	1	1	5	0.24	2.07

^a^ IF: Interference.

**Table 4:** NO_2_^−^ and AA^−^ contents in food samples detected by the developed method and the standard method.

Interference	Concentration (mg kg^−1^)			
	Standard Method ^a^		Development Method ^b^	
	AA^−^	NO_2_^−^	AA^−^	NO_2_^−^
Strawberry juice	258.90 ± 0.03	N.D.	246.57±0.10	N.D.
Guava juice	1213.47 ± 0.11	N.D.	1211.70 ± 0.53	N.D.
Green oak	13,210.00 ± 0.08	10.70 ± 0.02	13,441.48 ± 0.17	15.10 ± 0.00
Basil	13,737.36 ± 0.14	9.80 ± 0.01	13,693.33 ± 0.08	11.48 ± 0.00
Synthetic samples	435.00 ± 0.20	124.70 ± 0.00	436.80 ± 0.09	124.20 ± 0.02
Firilice	1667.72 ± 78.83	10.12 ± 7.50	1593.55 ± 221.39	9.20 ± 0.11
Bokchoy	755.81 ± 16.61	165.55 ± 4.56	733.42 ± 2.58	173.09 ± 0.48
Cos	2231.66 ± 11.69	43.23 ± 1.26	2212.30 ± 15.57	38.69 ± 1.17
Red oak	2627.24 ± 7.68	292.79 ± 4.09	2638.04 ± 3.45	276.26 ± 3.83
Butterhead lettuce	2335.36 ± 11.03	169.64 ± 1.00	2324.47 ± 8.69	164.88 ± 2.73
Red coral	1396.29 ± 12.74	171.79 ± 5.15	1400.62 ± 7.25	160.92 ± 3.81
Hydroponic nutrient	679.97 ± 6.23	38.55 ± 3.40	712.61 ± 4.84	56.69 ± 0.57

^a^ Standard method: UV–Visible spectrophotometric method. ^b^ Developed method: this work.

**Table 5:** A comparison of the electrochemical performance of the proposed electrode (CTS@Fe_3_O_4_/SPGNE) with previous studies to analyze AA^−^ and NO_2_^−^.

Electrode	Technique	Analyte	Sample	Linearity Range (μM)	LOD (µM)	Ref.
ZrO_2_@MWCNTs SPE	CA	NO_2_^−^	real food and water	5.0–100	0.94	[38]
Glassy carbon electrode (GCE)	CV ^b^	AA^−^	fruit juices and food supplement	2.5 × 10^3^–1 × 10^6^		[40]
Au nanoparticle/graphene–chitosan-modified electrode	CA ^a^	NO_2_^−^		0.9–18.9	0.30	[32]
Carbon SPE	CA ^a^	AA^−^	fruit juice	20–1000	0.70	[39]
a nanocomposite of polyneutral red and reduced graphene oxide paste electrode (pNR/rGO-PE)	CA ^a^	NO_2_^−^	food	0–14,000	0.017	[41]
Metallic copper Nanosheets/carbon paper electrode (Cu/CP)	CA ^a^	NO_2_^−^	drinking water	10–1000	0.079	[42]
Au-Cu_2_O/MWCNTs nanocomposite	DPV ^c^	AA^−^	biological samples	1–200	0.30	[43]
Fc(CO-Glu-Cys-Gly-OH) on screen-printed electrodes (Fc-ECG/SPE).	DPV ^c^	NO_2_^−^	pickle juice	1.0–50	0.30	[44]
GCE/CNO/oAP	CA ^a^	NO_2_^−^ and AA^−^	orange juice	0–50 (NO_2_^−^) and 0–50 (AA^−^)	0.82 (NO_2_^−^) and 0.34 (AA^−^)	[45]
GCE/CNO/thionine	CA ^a^	NO_2_^−^ and AA^−^	orange juice	0–50 (NO_2_^−^) and 0–50 (AA^−^)	1.89 (NO_2_^−^) and 0.66 (AA^−^)	[45]
CTS@Fe_3_O_4_/SPGNE	SWV ^d^	NO_2_^−^ and AA^−^	fruit juice and hydroponic vegetable	3–200 (NO_2_^−^) and 5–200 (AA^−^)	2.84 (NO_2_^−^) and 3.39 (AA^−^)	This work

^a^ CA: Chronoamperometry; ^b^ CV: cyclic voltammetry; ^c^ DPV: Differential Pulse Voltammetry; ^d^ SWV: square-wave voltammetry.

## Data Availability

Data are contained within the article and Supplementary Materials.

## Supplementary Materials

The following supporting information can be downloaded at: https://www.mdpi.com/article/10.3390/s25051431/s1, Figure S1 [47,48,49]. The optimum conditions for SWV parameters: (a) deposition time for a pre-preconcentration step, (b) frequency, (c) amplitude, and (d) step potential. Figure S2. (a) The cyclic voltammograms for analysis of phosphate buffer (pH 5) using the SPGNE (blue line) and the CTS@Fe3O4/SPGNE (red line) and (b) the SWV voltammograms for AA− and NO2− analysis in phosphate buffer (pH 5) using the SPGNE (blue line) and the CTS@Fe3O4/SPGNE (red line). Figure S3. Calibration plots under optimum conditions for (a) ascorbic acid (AA^−^) at a fixed 50 μM NO_2_^−^ and (b) nitrite (NO_2_^−^) at a fixed 50 μM AA^−^.
