# Supplementary material for: Application of Chitosan@Fe3O4 Nanoparticle-Modified Screen-Printed Graphene-Based Electrode for Simultaneous Analysis of Nitrite and Ascorbic Acid in Hydroponics and Fruit Juice
Source: Sensors (Basel). 2025 Feb 26;25(5):1431. doi: 10.3390/s25051431 (PMC11902332; doi:10.3390/s25051431)
Supplement: Supplementary file 1 [file sensors-25-01431-s001.zip › sensors-3448835-supplementary.pdf]

Supplymentary

1. the study of optimum conditions for SWV

The parameters of SWV such as deposition time in preconcentration step, frequency, amplitude and step potential were studied as shown in Figure A1. The obtained results show that pre-concentration step at potential -0.5 V for 15 s, scan potential -0.5 to 1.2 V, frequency 25 Hz, amplitude 25 mV s^{-1} and step potential 10 mV s^{-1} were used as optimum voltametric parameters.

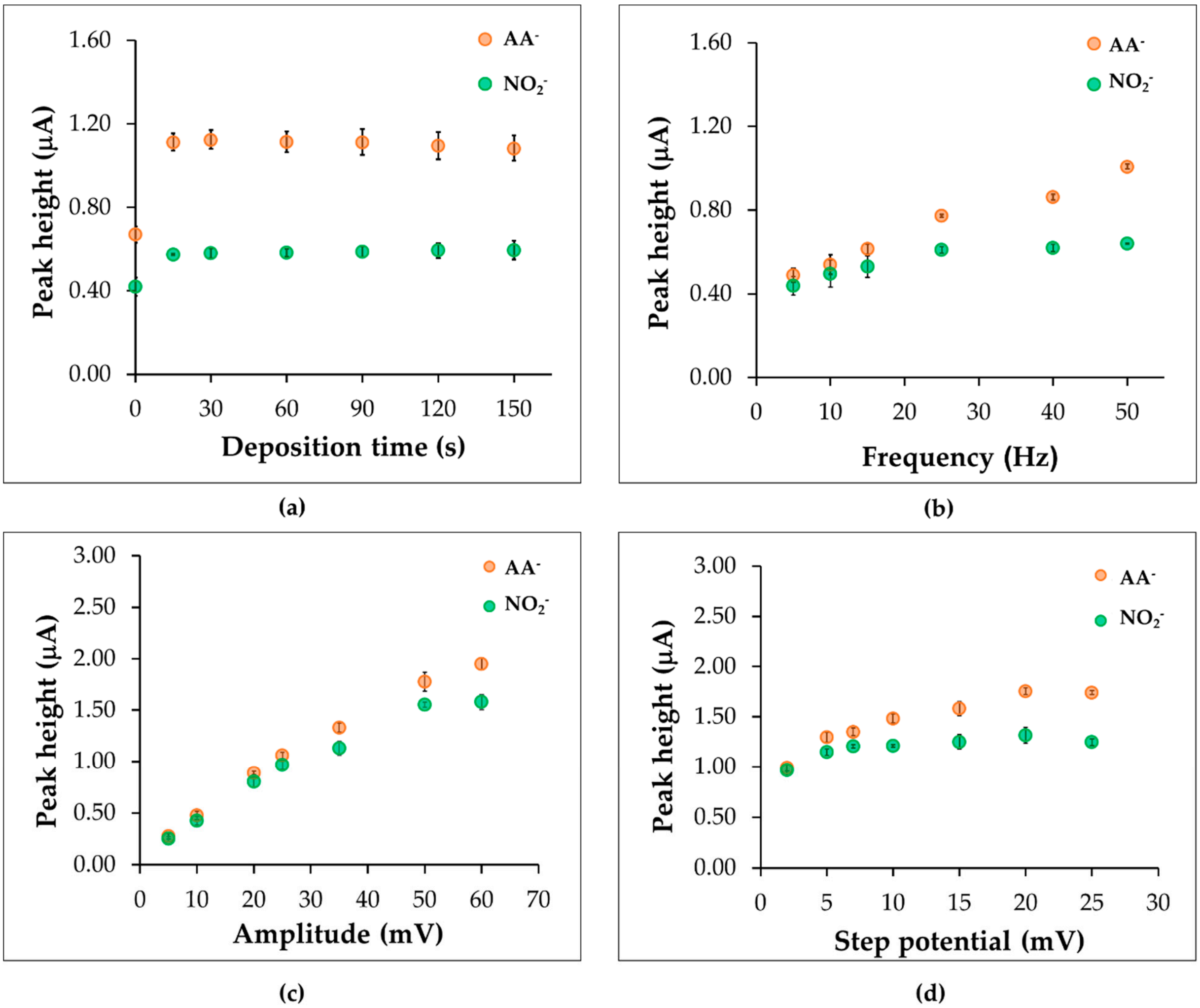

Figure S1. The optimum conditions for SWV parameters: (a) deposition time for pre-preconcentration step, (b) frequency, (c) amplitude and (d) step potential.

2. Preliminary study

2.1 Oxidation reactions of NO_2^- and AA^-

Previous observations in this study indicate that the oxidation potentials of NO_2^- and AA^- are similar to those reported in other researches, depending on the types of electrode material [47,48]. The oxidation reaction of NO_2^- to NO_3^- shown as follows:

$$NO_2^- + H_2O \longrightarrow NO_3^- + 2H^+ + 2e^- \quad \text{(b1)}$$

The oxidation potential of AA^-, it can vary slightly depending on the type of electrode as reported in a previous study [43,49], the oxidation reaction is as follows:

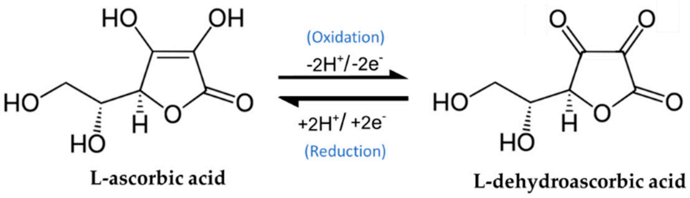

(b2)

2.2 Preliminary investigations for nitrite (NO_2^-) and ascorbic acid (AA^-) analysis

The cyclic voltammograms of phosphate buffer (pH 4) recorded using SPGNE exhibited a higher background current, as shown in Figure B1(a). Additionally, the simultaneous analysis of NO_2^- and AA^- using the SWV technique with SPGNE and CTS@Fe_3O_4/SPGNE was examined. The SWV voltammograms obtained using CTS@Fe_3O_4/SPGNE displayed suitable response signals (symmetric signal of AA^-), as illustrated in Figure B1(b). Therefore, CTS@Fe_3O_4/SPGNE was selected as the electrode for this study.

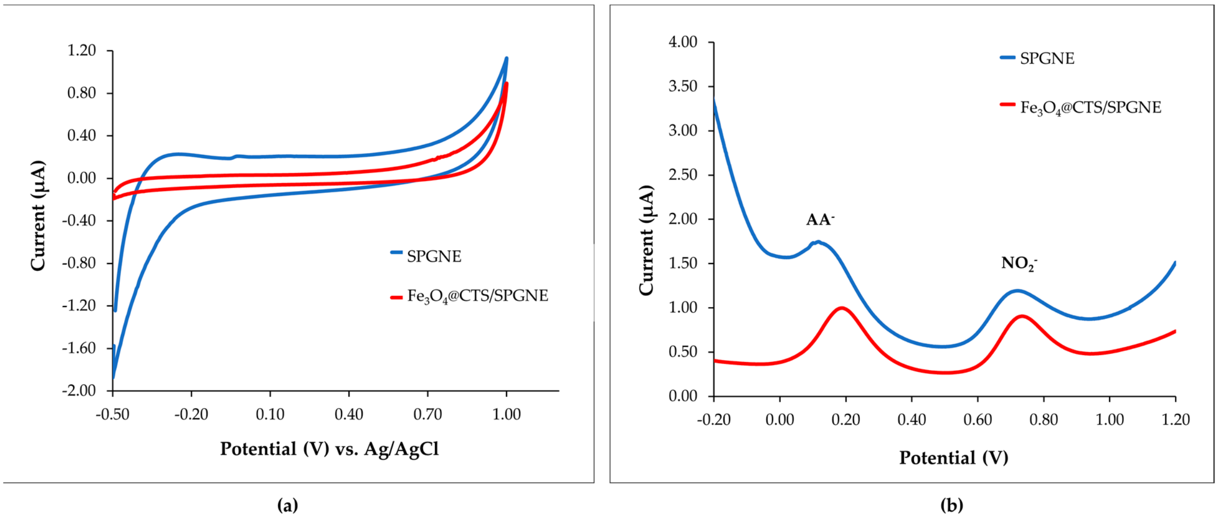

Figure S2. (a) The cyclic voltammograms for analysis of phosphate buffer (pH 5) using the SPGNE (blue line) and the CTS@Fe_3O_4/SPGNE (red line) and (b) the SWV voltammograms for AA^- and NO_2^- analysis in phosphate buffer (pH 5) using the SPGNE (blue line) and the CTS@Fe_3O_4/SPGNE (red line).

3. Linearity study

The calibration for AA^- at a fixed concentration of NO_2^- (50 µM) was studied. The responses observed that the NO_2^- signals were not influenced by increasing concentration of AA^- as shown in Figure C1. Moreover, the NO_2^- signal remained unaffected by the increasing concentration of AA^- (Figure C1(b)).

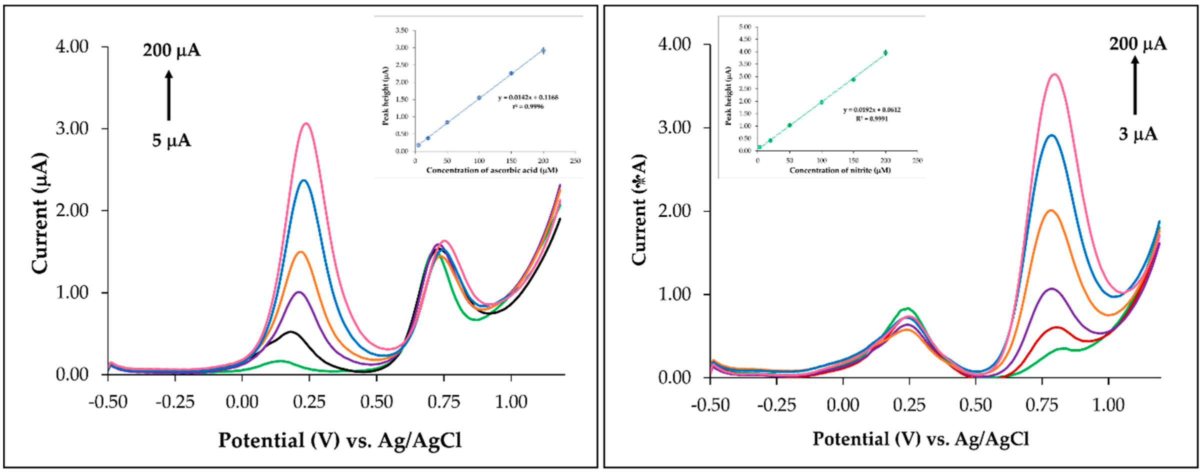

(a) **(b)**

Figure S3. Calibration plots under optimum conditions for (a) ascorbic acid (AA^-) at a fixed 50 µM NO_2^- and (b) nitrite (NO_2^-) at a fixed 50 µM AA^-

Reference

47. Pak, J.S.; Jang, P.H.; Pak, K.M.; Yang, W.C. Electrochemical Detection of Nitrite on PANI-TiO(2)/Pt Nanocomposite-Modified Carbon Paste Electrodes Using TOPSIS and Taguchi Methods. *ACS Omega* 2024, 9, 30583-30593, doi:10.1021/acsomega.4c02524.
48. Faisal, M.; Alam, M.M.; Ahmed, J.; Asiri, A.M.; Algethami, J.S.; Altholami, R.H.; Harraz, F.A.; Rahman, M.M. Efficient nitrite determination by electrochemical approach in liquid phase with ultrasonically prepared gold-nanoparticle-conjugated conducting polymer nanocomposites. *Front Chem* 2024, 12, 1358353, doi:10.3389/fchem.2024.1358353.
49. Amare, M. Electrochemical Determination of Ascorbic Acid in Pharmaceutical Tablets using Carbon Paste Electrode. *Organic & Medicinal Chemistry International Journal* 2019, 8, doi:10.19080/omcij.2019.08.555749.
